# Refining methodological approaches for analysing higher-throughput locomotor behaviour, using generalized additive mixed models

**DOI:** 10.3389/ftox.2026.1891859

**Published:** 2026-08-21

**Authors:** Gabrielle Wasser-Bennett, Sylvia Dimitriadou, Matthew J. Winter, A. Ross Brown, Courtney Hillman, Mhairi Miller, Charles R. Tyler

**Affiliations:** 1 Biosciences, University of Exeter, Exeter, United Kingdom; 2 Surrey Sleep Research Centre, Department of Clinical and Experimental Medicine, School of Biosciences, University of Surrey, Guildford, United Kingdom

**Keywords:** behaviour, ecotoxicology, light-dark, locomotion, new approach method (NAM), visual-motor response (VMR), zebrafish

## Abstract

**Introduction:**

Movement underlies most essential behaviours and is, therefore, frequently used as a behavioural endpoint in a wide range of phenotypic assessments. These include higher-throughput locomotor assays typically employing larval fish or occasionally invertebrate species, which are widely applied to assess the effects of chemical exposure or genetic modification on behaviour. Analysis of the resultant locomotor data, however, is challenging due to inter-individual variability, repeated measures (for each individual), and complex time-dependent responses. These challenges are further exacerbated when additional independent variables, such as light-dark cycling in the visual motor response (VMR) assay, are incorporated.

**Methods:**

Here we compare use of generalised additive mixed models (GAMMs) with conventional analysis of variance (ANOVA)-based approaches to assess chemical effects on locomotor behaviour, using a case study of the impact of clozapine exposure on (light/dark) in zebrafish embryo larvae.

**Results and Discussion:**

We show adoption of generalised additive mixed models (GAMMs) provides the most appropriate framework for analysing locomotor data, owing to their flexibility in modelling non-linear behavioural patterns, and their ability to explicitly account for dynamic inter-individual variability. While ANOVA identified a significant three-way interaction that precluded meaningful post hoc interpretation, its reliance on assumptions that are violated by these data limits the validity of the resulting inferences. In contrast, GAMMs accommodate the data structure and explain a greater proportion of the variance (59.7% compared to 54.1% for ANOVA), here revealing significant, light-dependent non-linear locomotor trajectories. GAMMs further identified treatment-specific effects, including reduced locomotion under light conditions across all clozapine treatments, and at the highest concentration only in dark conditions. Overall, our findings demonstrate that GAMMs offer high statistical rigour but with improved interpretability of treatment effects, supporting their wider adoption in analysing data from studies employing similar behavioural assessment paradigms.

## 1 Introduction

Locomotion is a key behavioural endpoint with clear ecological relevance and broad utility in *in vivo* toxicology and pharmacology (Boehmler et al., 2007; Wasser-Bennett et al., 2025). As an integrative readout of neuromuscular function, locomotor behaviour has been shown to be a sensitive proxy for neurotoxicity and has therefore become popular for assessing the behavioural impacts of chemical exposure in a range of species. This is particularly true for the zebrafish (*Danio rerio*), whose locomotor circuitry and developmental neurobiology are well characterised (Saint-Amant and Drapeau, 1998; Drapeau et al., 2002; Brustein et al., 2003). Furthermore, early maturation of their motor network allows chemical perturbations of early-life behaviours (particularly locomotion) to be interpreted in terms of known biological mechanisms, improving confidence in causal conclusions.

In addition to its physiological relevance, larval zebrafish locomotion is highly amenable to higher-throughput quantification. Automated video recording and tracking platforms enable simultaneous assessment of many individual larvae and are now well validated, if not standard, for the screening of behavioural endpoints after chemical exposure (Winter et al., 2008; Afrikanova et al., 2013; Hageter et al., 2025). Off the shelf software/hardware platforms, such as ViewPoint™, DanioVision™ and EthoVision XT™ (Supplementary Table S1) allow scalable, reproducible measurement of locomotor endpoints, facilitating large highly replicable experimental designs and the assessment of sizable compound libraries. Consequently, locomotion has become a widely adopted endpoint in larval zebrafish toxicological and pharmacological research (Rosa et al., 2022).

Despite the extensive use of such assays in chemical screens and resultant evidence of [neuroactive] chemical induced effects on locomotion (Boehmler et al., 2007; Irons et al., 2010; Gundlach et al., 2022), behavioural data typically exhibit considerable variability, limiting interpretability (Hill et al., 2023; Wasser-Bennett et al., 2025). The application of controlled environmental stimuli can reduce such baseline variability and enhance detection of distinguished treatment-specific effects (Fitzgerald et al., 2019; 2021; MacPhail et al., 2009). Among these, light–dark transition paradigms are particularly effective in eliciting robust and stereotyped behavioural responses (Richendrfer et al., 2014; Sztal et al., 2016; Kokel et al., 2010), thus, increasing the precision with which deviations from stereotyped swimming behaviour (e.g., following neuroactive drug treatment) are quantified. The larval light–dark transition assay, commonly referred to as the visual motor response (VMR), measures locomotion during repeated cycles of illumination and darkness. Larvae are generally organised in well-plates and isolated within an enclosed environment, following which abrupt changes in illumination (100% light-to-100% darkness) at set frequencies evoke rapid shifts in activity that reflect internal arousal states and changes in sensory processing (Padilla et al., 2011). The VMR has, therefore, become a widely used method for investigating the behavioural impacts of neuroactive chemicals, including pharmaceuticals such as antipsychotics (Bruni et al., 2016; Huang et al., 2019; Oliveri and Levin, 2019), stimulants (Irons et al., 2010; Maeda et al., 2021; Hillman et al., 2024b) and anti-/pro-convulsant drugs (Zellner et al., 2011; Ellis et al., 2012; Peng et al., 2016).

Locomotor metrics in the VMR are typically summarised as distance or speed travelled during defined light and dark periods, and results can then be compared across treatments. Larvae typically exhibit low activity during the light phase, and heightened activity following abrupt transitions to darkness, a pattern associated with increased arousal and light-seeking exploratory behaviour (Burgess and Granato, 2007; Burgess et al., 2010; Basnet et al., 2019). While numerous endpoints can be extracted from VMR datasets (Rowson et al., 2025), increasing metric complexity does not necessarily improve inferential power. Over-fragmentation of behavioural readouts risks obscuring coherent treatment effects, particularly when applied statistical frameworks do not faithfully reflect the structure of the underlying dataset.

**FIGURE 1:**
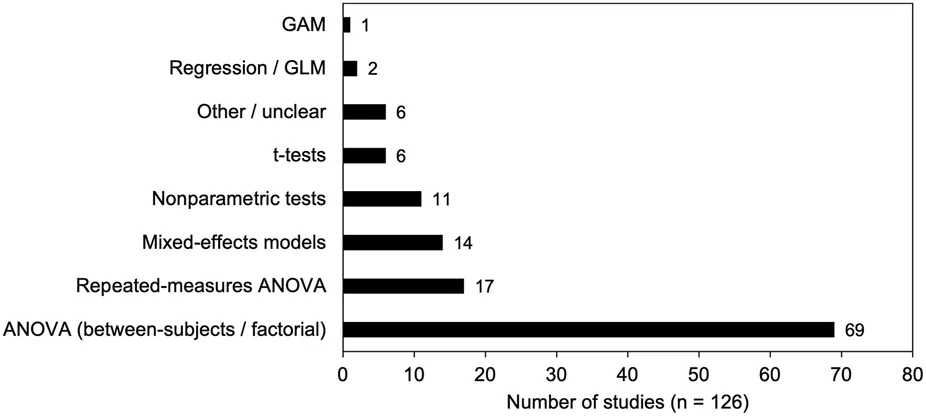
Frequency of use of statistical frameworks for larval zebrafish light–dark locomotor studies (*n = 126*). Studies were classified according to the statistical framework used for data analysis. Where multiple statistical tests were reported, classification was based on the highest-order model used for primary inference. One-way and multi-factor analysis of variance ANOVA designs were grouped as ‘between-subjects ANOVA’. A detailed comparison of experimental parameters and statistical analyses is provided in Supplementary Table S1 (xlsx). GAM–General Additive Model; GLM–General Linear Model. To our knowledge, there are no existing examples of mixed models (GLMMs or GAMMs being used for VMR studies.

Despite the relative simplicity of data collection, analysis of VMR datasets is inherently challenging due to the generation of longitudinal, cyclic (given the light-dark stimuli), and highly structured time-series data. Statistical approaches used for VMR data in the literature frequently reduce this complexity using data transformation, however this approach risks reducing statistical rigour, as well as masking random effects, natural variation and inter-individual variability (Liu et al., 2015; Liu et al., 2017; Gauthier and Vijayan, 2018; Rowson et al., 2025). Here, our systematic comparison of published larval zebrafish light–dark locomotor studies revealed substantial heterogeneity in experimental design and analytical practice (Figure 1; Supplementary Table S1). Although repeated light–dark transition paradigms predominate, the temporal structure of these assays varies widely, with lighting-epoch durations ranging from ≤1 min to >600 min (Supplementary Table S1). This variability complicates direct comparison of behavioural outcomes across studies and may contribute to reported inconsistent sensitivity of locomotor responses to chemical perturbation.

Most statistical approaches currently used for VMR analysis (Figure 1; Supplementary Table S1) are not well suited to behavioural time-series data. Studies often rely on analysis of variance (ANOVA)-based approaches, with comparatively fewer studies employing repeated-measures or mixed-effects models (Figure 1; Supplementary Table S1). In many published studies, the description of key statistical practices is also limited, with assumption testing, effect size estimation, and model diagnostics infrequently reported. In some cases, the temporal structure and complexity of the assay is effectively discarded by averaging locomotion across the entire recording period, yielding a reductive summary that masks dynamic behavioural trajectories that could be modified by exposure to the chemical being assessed. These approaches do not currently address the fundamental issue that VMR data are inherently non-linear, heteroscedastic, and temporally dependent (Liu et al., 2015; Liu et al.,2017). Even more advanced mixed-model approaches such as generalised linear mixed model (GLMM) implementations, similarly, sacrifice quantitative information by reducing continuous movement variables to categorical states (binarization: moving vs. not moving), thereby diminishing analytical resolution (Liu et al., 2017). To overcome such issues, Gauthier and Vijayan (2018), suggest a more advanced linear mixed model. However, to date this has only been tested for an experimental design of limited complexity, which does not fully recapitulate the complex light cycling used during a typical VMR assessment. Collectively, these observations highlight the need for greater methodological standardisation and more appropriate analytical frameworks that explicitly account for behavioural dynamics across repeated light–dark transitions, as well as those related to altered chemical concentration.

With this in mind, we, therefore, suggest an approach that allows for the modelling of non-linear relationships between the dependent and independent variables without the need for transformation or binarization of data as being appropriate. Generalised Additive Mixed Models (GAMMs) provide a flexible solution by capturing smooth, non-linear relationships between predictors (e.g., within light and dark phases) and behavioural responses while accommodating random effects that account for repeated (non-independent) measures and inter-individual variability (on the same individuals over multiple contexts), thereby handling within-subject correlation (Hastie and Tibshirani, 1986; Hastie and Tibshirani, 2017). GAMMs enable simultaneous modelling of light-phase, dark-phase, and transition dynamics, as well as treatment-by-illumination interactions, preserving the full temporal richness of the dataset, without dataset compression and potential loss of biological granularity.

Using a typical experimental VMR dataset derived from zebrafish larvae exposed to the antipsychotic drug clozapine at 4 days post fertilisation (dpf), we employ several established statistical approaches currently used in the analysis of larval zebrafish VMR data and compare them with an alternative GAMM-based framework. By quantifying how different analytical strategies facilitate or limit inference, we aim to identify strengths and weaknesses of prevailing methods and to provide practical guidance to researchers for selecting statistically appropriate tools for their own analyses. Establishing more rigorous and standardised analytical practices will improve reproducibility, enhance cross-laboratory comparability, and strengthen the interpretive power of the widely used VMR assay.

## 2 Materials and methods

### 2.1 Ethics statement

All experimental procedures were conducted in accordance with the United Kingdom Animals (Scientific Procedures) Act (1986) and the University of Exeter’s Animal Welfare and Ethical Review Body. All animals used in this study were classified as being at a pre-protected stage <5 dpf (i.e. 118 hpf) according to ASPA.

### 2.2 Experimental animals and husbandry

Zebrafish (*Danio rerio*) of the Wild Indian Karyotype (WIK) strain, maintained at the University of Exeter Aquatic Resource Centre, were used for embryo generation. Adult zebrafish were housed in 8 L tanks at a stocking density of 5 individuals per L (sex ratio ∼1:1), maintained at 28 (+/-1) °C under a 12 h:12 h light/dark photoperiod with 20 min dusk-dawn transition periods. Fish were fed twice daily with commercial flake food (Zebrafeed, Sparos) supplemented with live *Artemia nauplii*. Freshly spawned embryos were collected immediately and pooled in fish dilution water (dilution water control; DWC, consisting of dechlorinated mains tap water filtered by reverse osmosis and subsequently reconstituted with Analar-grade mineral salts to achieve a standard synthetic freshwater composition).

Following assessment of fertilisation success and embryo quality, embryos were randomly allocated (*n* = 20 per group) into round glass crystallization dishes (350 mL capacity) maintained at a volume of 200 mL of exposure solution (clozapine treatment, solvent control, or dilution water control [DWC]). Embryos were reared in groups to permit social interaction while avoiding crowding stress, conditions that more closely reflect natural zebrafish behaviour and have been shown to improve the robustness of early larval phenotypes (Suriyampola et al., 2015).

Chemical exposure commenced at 2 h post fertilisation (hpf), prior to the onset of blastulation, and continued until behavioural testing at 4 dpf. Embryos were incubated under static conditions at 28.5 °C under a 14:10 h light/dark cycle. Embryos were inspected daily, and any non-viable individuals were promptly removed and euthanized via an overdose of anaesthetic.

### 2.3 Clozapine preparation and dosing

Clozapine (CAS No. 5786-21-0; purity <97%) was obtained from Fisher Scientific. Stock solutions were prepared in dimethyl sulfoxide (DMSO), which were then stored at 4 °C for up to 3 months. DMSO is a widely used solvent with zebrafish embryo chemical exposures with concentrations up to ≤1% v/v showing no physiological effects (de Oliveira et al., 2021). On the day of exposure, stock solutions were freshly diluted in dilution control water to achieve the desired nominal concentration, with a final solvent concentration of 0.1% (v/v) DMSO present across all treatments. Equivalent solvent controls (SC; 0.1% DMSO in DWC) and dilution water controls (DWC only) were assessed alongside treated animal groups in all experiments.

### 2.4 Exposure concentration range

The behavioural exposure series was defined using a sublethal nominal no observed effect concentration (NOEC; body length) determined during preliminary range-finding experiments. The experiments were conducted in accordance with a modified OECD Fish Embryo Toxicity (FET) Test guideline (OECD 236). All clozapine test concentrations used in this study were below those previously reported in the literature to elicit adverse effects in zebrafish embryo-larvae (Zhang et al., 2021; Lee et al., 2013). A total of four clozapine exposure concentrations (0.1–6.25 μM) were tested together alongside solvent and DWC controls.

### 2.5 Locomotor behavioural assay

**FIGURE 2:**
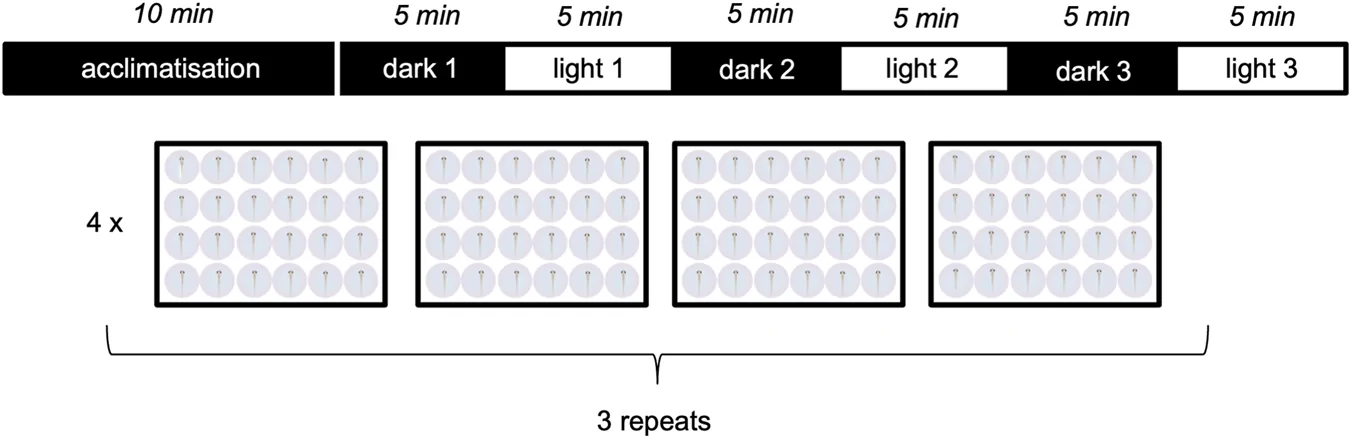
Experimental schematic of the larval visual motor response (VMR) assay. Following an acclimatisation period, larvae were exposed to three consecutive dark–light cycles. Each experiment is represented by 4 x 24-well plate (*n = 16* per treatment), and this was repeated 3 times (*n = 48* in total/treatment).

Larval locomotor activity was assessed using an automated four-camera videotracking system (VideoTrack v2.5, Viewpoint, France; Gould et al., 2024). For this, 4 dpf, larvae were individually transferred in test solution into wells of a 24-well plate (Corning Incorporated, United Kingdom). For each compound, treatments were distributed equally across four plates, yielding 16 larvae per treatment per experimental run (with three experimental runs in total yielding *n =* 48 per treatment; Figure 2). Behavioural testing and data collection commenced at approximately 10:00 a.m. (≈118 hpf) and was completed by 12:00 p.m., (120 hpf).

Larval activity was recorded for 40 min under alternating light (white light ca. 500 Lux) and dark (recorded under infrared illumination) conditions. The protocol comprised a 10-min light acclimation period, followed by six abrupt 100% light/dark transitions, each lasting 5 min. This protocol was designed to reliably stimulate locomotor activity in relatively quiescent larvae and to provide a mild anxiogenic challenge (Schnörr et al., 2012; Hillman et al., 2024a). From the recorded videos, total distance travelled was quantified in 60 s integration intervals.

On completion of behavioural testing, larvae were terminated by overdose of anaesthetic in a static bath immersion (benzocaine, 2000 mg/L in 5% ethanol) followed by destruction of brain in accordance with the local rules and standard operating procedures.

### 2.6 Statistical analysis

All tracking data from replicate plates were pooled for statistical analysis. Statistical analyses were performed using R version 4.5 (R Core Team, 2025) to compare treatment effects on normalized movement (% control) over time under alternating dark and light. Total distance travelled by individual larvae was calculated for a 30-min tracking period excluding the acclimatisation period (comprising the initial 10 min). The time period analysed comprised three 5-min alternating periods of light and dark periods (Hillman et al., 2024a).

#### 2.6.1 Normalisation

Data were normalised prior to any statistical analysis. The variability of behavioural responses in larval zebrafish is well documented (e.g., Jacobs and Ryu, 2023), while the VMR specifically has been shown to differ between different batches (Xie et al., 2019; Hillman et al., 2024b), times of the day, and laboratories (Hillman et al., 2024b). Data normalisation has been shown to reduce some of the data variability introduced by genetic and environmental factors such as batch-to-batch variability (Xie et al., 2019). Importantly, data normalisation is often the first step in data analysis when traditional statistical analyses assuming normal distributions of residuals (such as ANOVA) are undertaken (Xie et al., 2019). The normalisation approach used here follows that detailed by Hillman et al., (2024a), where movement per minute per fish was normalised to a baseline (compared to locomotion in controls). A solvent-control baseline was defined by pooling all vehicle-treated fish and calculating the total mean locomotion across the entire 30 min assay (post-acclimatisation period). Every raw observation was then converted to a percentage of this control mean according to:

$${ND}_{t,f}=100\cdot \frac{{Dist}_{t,f}}{{Dist}_{control}}$$

Where:

${ND}_{t,f}$ is the normalised distance of fish f at minute t ,

${Dist}_{t,f}$ is the distance travelled by fish f during minute t , and

${Dist}_{control}$ is the control distance.

To minimise the impact of tracking artefacts, at each minute and within each treatment, the group mean and standard deviation was computed and any values lying more than 3 standard deviations from their group mean were considered outliers and removed (Howell, 1998; Leys et al., 2013). The percentage of outliers detected was similar across all levels of experimental treatments (0.9%–1.3% for clozapine-exposed animals, 0.4% for solvent control, and 1% for water control). To ensure that outlier removal does not affect data interpretation, we also carried out the same statistical analysis (but in a smaller scale, focusing only on GAMM approaches) using the same dataset including outliers, and confirmed that the results obtained are very similar in both instances (see Supplementary Material). All subsequent statistical analysis and graphical summaries (mean ± SEM) were performed on these baseline-normalised, outlier-filtered data.

#### 2.6.2 Line plot visualisation

Time-series plots of normalized movement (% control) were generated in ggplot2 R package (Wickham, 2016) as mean ± SEM over 30 min. Alternating grey (dark) and white (light) 5-min background rectangles delineate illumination phases; treatment groups were overlaid as coloured lines and points with vertical error bars (mean ± SEM).

#### 2.6.3 Analysis of locomotor behaviour

Data were analysed by fitting several different statistical models (the full list is provided in Table 1, with additional details in the Supplementary Material; Supplementary Table S1). Analysis of variance (ANOVA)/linear modelling was undertaken using base R (R Core Team, 2025), while generalised additive (mixed) effects models (GAMs and GAMMs) were run using the *mgcv* R package (v1.9-1; Wood, 2011). The latter included the use of a thin-plate smoother, with k = 30, which was then validated separately using k-fold cross validation (Hijmans, 2012; see Supplementary Material).

**TABLE 1:** Summary of the main statistical approaches for VMR data analysis used in this paper, alongside their respective advantages and disadvantages (specifically for use with VMR datasets).

Type of statistical analysis	Advantages	Disadvantages
Analysis of variance (ANOVA) and related approaches	- Quick and easy to run - Accessible - Can provide treatment contrasts/*post hoc* analysis (depending on whether time is treated as a continuous or categorical variable)	- Not applicable to repeated-measures designs (should be using repeated-measures ANOVA instead) - Other core assumptions are often violated - If time is included as a continuous variable/covariate (ANCOVA) then interpretation of any *post hoc* comparisons is difficult
Linear mixed effects models (LMER)	- Quick and easy to run - Accessible - Can handle repeated-measures designs - Can provide treatment contrasts/*post hoc* analysis (depending on whether time is treated as a continuous or categorical variable)	- Core assumptions often violated, particularly the assumption of linearity of residuals - Requires more extensive validation to choose the appropriate random effect structure - If time is included as a continuous variable/covariate then interpretation of any *post hoc* comparisons is difficult
Generalised linear models (GLM)	- Relatively accessible (although model optimisation is needed) - Can handle non-normal residual distributions - Can provide treatment contrasts/*post hoc* analysis (depending on whether time is treated as a continuous or categorical variable)	- Not applicable to repeated-measures designs - Requires more extensive model validation to choose the appropriate distribution and link function - May present with overdispersion issues - If time is included as a continuous variable/covariate then interpretation of any *post hoc* comparisons is difficult
Generalised mixed effects models (GLMER)	- Can handle repeated-measures designs - Can handle non-normal residual distributions - Can provide treatment contrasts/*post hoc* analysis (depending on whether time is treated as a continuous or categorical variable)	- Less accessible/not widely available across statistical software packages - Can be computationally expensive, depending on the model structure - Requires more extensive model validation to choose the appropriate distribution and link function, as well as random effects structure - May present with overdispersion issues - If time is included as a continuous variable/covariate then interpretation of any *post hoc* comparisons is difficult
Generalised additive mixed effects models	- Can handle repeated-measures designs - Can handle non-normal residual distributions - Can model complex relationships between different factors - Due to their flexibility, their core assumptions are less extensive - Can provide easily interpretable/biologically meaningful *post hoc* comparisons	- Not widely implemented across statistical software/less accessible - Computationally intensive - Requires extensive model validation to choose the appropriate distribution and link function, smoothing parameters, as well as random effects structure - Fewer resources available to aid implementation

The linear model included the total distance travelled (in mm), time (excluding the acclimation period), treatment (a factor with 6 levels including the control condition), and the lighting regime (Light/Dark), as well as a 3-way interaction between these parameters. The GAMMs included the total distance travelled (in mm), the lighting regime, the treatment, and a smooth for time for each level of the lighting condition. More details on the validation of the final GAMM specification are provided in the Supplementary Material (including details on k validation and the selection of random effects). The optimal GAMM had a Gaussian family with a log link function. The nested random effects were individual fish ID to account for any differences between individual larvae, and trial to account for any variation between different experimental effects, with fish ID nested within trial. The residuals of all models were inspected visually to assess model fit, using the DHARMa R package v0.4.7 (Hartig, 2025). Instances where model fit appeared suboptimal are noted in the results section (additional information including all diagnostic plots are available in the Supplementary Material). Where a statistically significant interaction between model variables was observed, *post hoc* comparisons against the control treatment were undertaken using the emmeans v 1.10.3 R package (Lenth et al., 2018), allowing for Dunnett’s adjustment of p values.

In order to formally assess whether there is a non-linear relationship between Time and the other model parameters, we fitted a GAM (with the same structure as the GAMM but excluding the random effects) with the Restricted Maximum Likelihood Method, to allow for comparison to the linear model. Competing models were compared using Akaike’s Information Criterion (AIC), with lower AIC values indicating better support; models differing by more than 2 AIC (ΔAIC>2) units were considered meaningfully different in support. All GA(M)Ms were fitted using the mgcv R package (Wood et al., 2016; Wood, 2017) with Restricted Maximum Likelihood (REML) smoothing parameter estimation; AIC was computed via the AIC function, which uses effective degrees of freedom, accounting for penalisation and smoothing parameter uncertainty (Wood et al., 2016; Wood, 2017), reducing the tendency of conditional AIC to over-select highly complex models. AIC values are therefore directly comparable between GA(M)Ms and the linear models, given that the model terms remained the same.

## 3 Results

**FIGURE 3:**
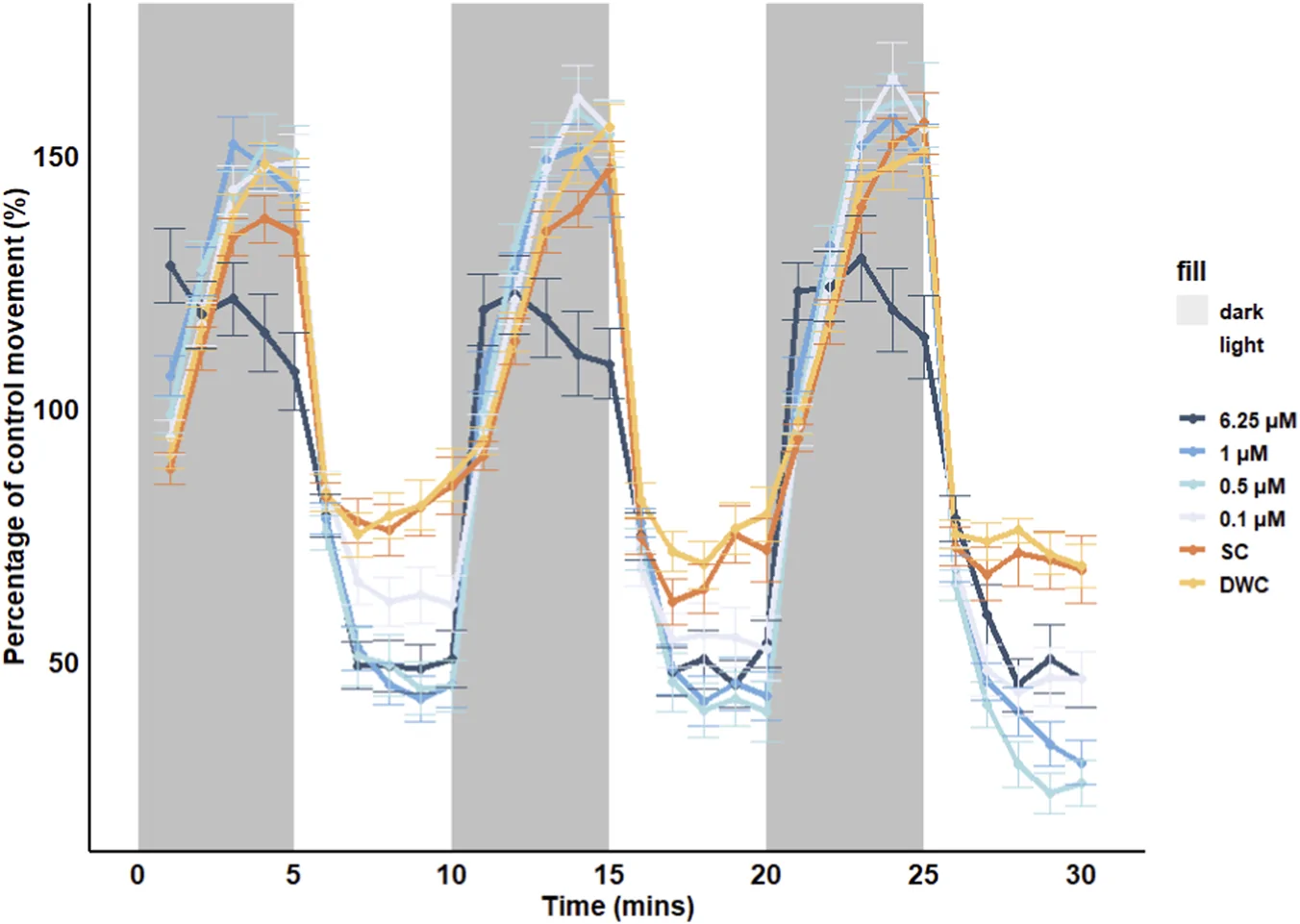
Locomotion in zebrafish embryo-larvae exposed to clozapine treatment (normalised distance moved) under repeated light-dark transitions. 30-min time course of locomotor activity normalised (as %) to the average overall movement of solvent control (SC) animals. Data are shown as group (*n =* 48) means (±SEM). Grey bands represent dark (no illumination), and white light (illuminated) phases. The colours of the lines denote clozapine concentrations. The solvent (dimethyl sulfoxide - DMSO) was used at 0.1% v/v in the preparation of clozapine treatments and the SC.

Exposure to the neuroactive pharmaceutical clozapine (0.1–6.25 μM) induced concentration-dependent changes in larval zebrafish behaviour as measured in the VMR assay, with total distance travelled differing significantly between test concentrations (Figure 3). Multiple modelling approaches were evaluated to analyse for treatment effects. Among these, ANOVA provided the only model with acceptable fit and stability and is therefore presented as the primary analytical framework against which the GAMM is evaluated. ANOVA was also prioritised because it remains the most widely used and recognisable approach in for light–dark locomotor studies (Figure 1). Alternative models, including General Linear Models (GLMs) and Linear Mixed Effects Regression (LMER), exhibited substantial fit violations and heteroscedasticity, while combined GLMER models failed to converge due to singularity issues. Full details of these additional analyses are provided in the Supplementary Material (Supplementary Table S1).

### 3.1 The linear model/ANOVA approach

Analysis of the dataset using ANOVA/linear model methods showed a significant 3-way interaction between the time elapsed, light condition (Light/Dark), and treatment (clozapine concentration) (F_(5, 8196)_ = 9.054, p < 0.001). The model explained 54.1% of the total variance (R^2^ = 0.541). Given that time (a continuous variable) is included in a statistically significant 3-way interaction, direct comparisons between different lighting regimes (light/dark) and treatment levels (clozapine concentrations) across the duration of the assay are not possible. When looking at the combined effect of light and dark and clozapine on the effect (trend or slope) of time, i.e., how this combined effect varies across the duration of the trial, *post hoc* analysis suggested a difference in the effect (slope) of time for 6.25 µM compared to the control condition, but only in the dark (slope estimate: -0.953, t = −4.711, p < 0.001) (Table 2).

**TABLE 2:** The influence of lighting conditions and clozapine exposure on the effect (trend/slope) of time, during a standard locomotion assay compared to the solvent control.

Lighting condition	Treatment (µM)	Estimate	Std. error	t-value	p-value
Light	SC	​	​	​	​
​	6.25	0.470	0.202	2.329	0.0823
​	1	−0.032	0.202	−0.160	0.9977
​	0.5	−0.314	0.202	−0.160	0.3867
​	0.1	−0.251	0.202	−1.246	0.5806
​	DWC	0.257	0.203	1.625	0.5680
Dark	SC	​	​	​	​
​	**6.25**	**−0.953**	**0.202**	**−4.711**	**<0.001**
​	1	−0.448	0.203	−2.203	0.1111
​	0.5	0.057	0.203	0.283	0.9894
​	0.1	0.009	0.202	0.046	0.9999
​	DWC	−0.329	0.202	−1.626	0.3454

Data were analysed using a linear model (ANOVA). Bold denotes statistically significant differences compared to the solvent control (p values after Dunnett’s adjustment). A DWC (water control) was included to assess the effect of solvent (0.1% DMSO) exposure compared to normal zebrafish culture water and was found not to have an effect. All treatment levels were compared to the SC.

Alternatively, *post hoc* analysis can focus on the contrasts between light/dark and the different concentrations of clozapine for specific time points (e.g., at the middle or end of each light/dark period). Although this approach can yield several statistically significant differences between the different experimental groups and lighting conditions, the choice of time points would be arbitrary and would affect the results: as seen in Supplementary Results (Supplementary Tables S2, S3), changing the timepoints selected for comparisons can drastically affect the results obtained from such analysis. This discrepancy of results depending on the choice of timepoints for analysis further demonstrates that, as shown in Table 2, the direction (i.e., slope) of the overall effect of Time differs between light and dark, as well as the different clozapine concentrations tested here.

It should be noted that several of the core assumptions of ANOVA models were violated by the model presented here, most notably the assumption of independence of observations (given that the dataset contains repeated measurements from the same individuals), and the homogeneity of regression slopes, given that we found a statistically significant interaction between time, clozapine administration, and the lighting regime.

**FIGURE 4:**
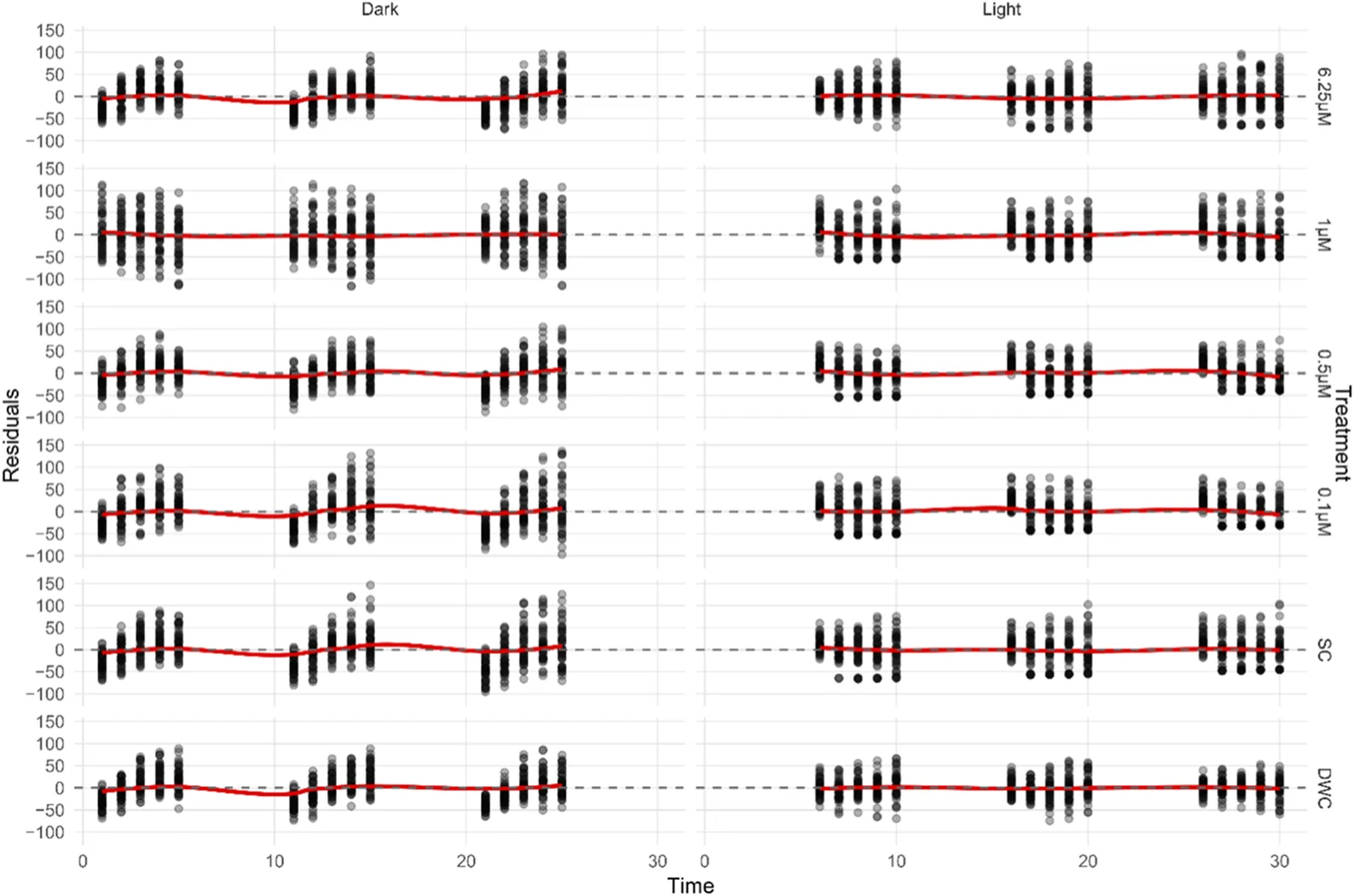
Residuals of the Analysis of Variance (ANOVA)/linear model plotted against Time, separately for Light and Dark conditions (left and right panels, respectively) and each clozapine treatment level (top: 6.25 μM of clozapine, bottom: water control). If the relationship between Time and the rest of the variables was linear, the residuals would be evenly dispersed around zero; however, in this case, there are clear, systemic curved patterns as shown by the trend (red) line. This curvature is particularly evident in the Dark condition (left panel), confirming that the relationship between time and the other model variables is not linear. Abbreviations used: SC, solvent control; DWC, distilled water control.

Visual inspection of the data (Figure 3) suggested a non-linear relationship between zebrafish larval locomotion and lighting condition with time, and this was further corroborated by plotting the model residuals of time for each level of treatment and light/dark phase (Figure 4). A comparison using Akaike’s Information Criterion (AIC) between the linear ANOVA model used above, and a generalised additive model (GAM) with the same parameters confirmed that allowing for a non-linear, light condition-specific smooth for time provides a substantially better fit (ANOVA: AIC = 81,507.5, df = 25.0; GAM: AIC = 80,428.8, edf = 36.7; ΔAIC = 1,078.8, in favour of the GAM). The GAM also explained more total variance (R^2^ = 0.598) than the ANOVA.

### 3.2 The GAMM approach

Analysing zebrafish larvae locomotion data (Figure 3) using a GAMM approach demonstrated a statistically significant interaction between the lighting conditions and treatment (clozapine concentration) (F_(10.9, 8185)_ = 171.06, p < 0.001). This suggested that the response of larvae to the change in lighting conditions is affected by exposure to different concentrations of clozapine. More specifically, in the dark phase, only the highest concentration (6.25 µM) led to a statistically significant decrease in locomotion compared to the control (SC) condition (estimated effect on the log scale: -0.0946, t = −2.56, p = 0.054) (Table 3). In the light phase, all clozapine treatments (0.1–6.25 µM) led to a decrease in total distance travelled compared to the control condition (see Table 3), while the DWC led to an apparent marginal increase which did not reach statistical significance (estimated effect on the log scale: 0.0929, t = 2.33, p = 0.083). The time smooths for Light and Dark conditions were both significant (Dark: F_(12.7, 8185)_ = 116.3, p < 0.001; Light: F_(10.9, 8185)_ = 31.7, p < 0.001), confirming non-linear trajectories dependent on the lighting condition. The GAMM explained 59.7% of variance (R^2^ = 0.597).

**TABLE 3:** Estimated effect of lighting conditions and clozapine exposure on the total distance travelled, during a standard locomotion assay compared to the solvent control (SC: DMSO 0.1% v/v).

Lighting condition	Treatment (µM)	Estimate	Std. error	t-value	p-value
Light	SC	​	​	​	​
​	**6.25**	**−0.3483**	**0.0426**	**−8.183**	**<0.001**
​	**1**	**−0.4061**	**0.0434**	**−9.364**	**<0.001**
​	**0.5**	**−0.5202**	**0.0447**	**−11.646**	**<0.001**
​	**0.1**	**−0.2394**	**0.0418**	**−5.733**	**<0.001**
​	DWC	0.0929	0.0399	2.327	0.0829
Dark	SC	​	​	​	​
​	**6.25**	**−0.0946**	**0.0370**	**−2.561**	**0.0454**
​	1	0.0798	0.0366	2.182	0.1165
​	0.5	0.0893	0.0366	2.438	0.0627
​	0.1	0.0740	0.0365	2.026	0.1645
​	DWC	0.0405	0.0365	1.107	0.6696

Data were analysed by a generalised additive mixed model (GAMM). Bold denotes statistically significant differences compared to the SC (p values after Dunnett’s adjustment). A DWC, was included to assess the effect of solvent (DMSO, 0.1% v/v) exposure compared to normal culture water and was found not to have an effect.

## 4 Discussion

This study compared the performance of different statistical approaches in the analysis of how clozapine exposure affects locomotor behaviour in 4dpf larval zebrafish, using the well-established VMR assay. We demonstrate that GAMMs capture the temporal complexity and dynamic structure of larval responses to light/dark cycling and improve the resolution with which chemical-induced locomotor phenotypes can be detected within this assay. The data analysed using the GAMM revealed that clozapine administration affects overall locomotion, and that this effect is dependent on lighting conditions. Notably, the highest concentration (6.25 µM) produced a significant reduction in distance travelled under both lighting conditions.

To benchmark the performance of GAMM analysis versus conventional analytical practices, a standard ANOVA was first applied to the dataset, given its most frequent use in the analysis of VMR data in the published literature. When analysed in this way, the data revealed a statistically significant three-way relationship between time, clozapine administration, and lighting regime, suggesting that locomotor behaviour differed between treatment groups depending on the (light/dark) illumination context and time elapsed. However, *post hoc* comparisons did not reveal any meaningful differences between experimental conditions. This limitation arises from the inclusion of time, a continuous variable, within a three-way interaction. Although the *post hoc* analysis indicates that the time-dependent slope, and therefore its overall effect, differs between the highest clozapine concentration (6.25 μM) and the control group in the dark phase, this distinction is challenging to interpret in a biologically meaningful way. More specifically, these findings suggest that the effect of time differs between the highest concentration of clozapine and the solvent control condition in the dark, but not the light phase. In essence, in each dark phase, as time passes, fish which were administered the highest concentration of clozapine move less, while fish which were administered the solvent control move more. While this conclusion may provide some insight into the effects of time, and therefore habituation, during the dark phases, it provides little information about the overall effects of clozapine on locomotion. One possible workaround would be to remove the three-way interaction from the model and treat time as a separate factor. However, doing so would impose the assumption that the interaction between lighting regime and clozapine concentration remains constant throughout the trial, thereby ignoring the context-dependent dynamics that the comparison to a GAM suggests are present (Duncan and Kefford, 2021). The inclusion of individual fish ID as a random factor (linear mixed effects model – LMER) to account for repeated measures and inter-individual variation marginally improved the amount of variance explained by the model, as indicated by the difference between the respective R-squared values (R^2^). Similar to the ANOVA model, the LMER also showed a statistically significant three-way interaction between time, light/dark, and clozapine concentration, as did a comparable generalised linear model (GLM) allowing for both continuous/categorical predictors and non-normal data (see Supplementary Material, Supplementary Table S1). Nevertheless, diagnostic evaluation indicated that neither the ANOVA or GLM was well fitted or able to capture the structure of the data, reinforcing their limitations for this type of behavioural data.

Incorporating GAMMs to account for the non-linear relationship between time and the other model variables revealed statistically significant smooths for time across both light and dark conditions, confirming that locomotor trajectories are non-linear and lighting status dependent. Within this analytical framework, clozapine exposure significantly affected the behavioural responses of larvae to alternating illumination, indicating that treatment effects on activity occur dynamically. The inclusion of time in the GAMM, while accommodating different smooths for dark and light conditions allows for a more flexible model compared to ANOVA and other tested linear modelling approaches, while simultaneously preserving statistical interpretability with meaningful *post hoc* comparisons. Unlike linear three-way interactions involving continuous variables, this structure enables relevant comparisons between specific levels of treatment groups and the control condition, without collapsing the temporal complexity of the dataset. Crucially, all statistical analysis techniques are only valid if a certain set of assumptions about the properties of the data and the relationship between the various factors are met (such as the normal distribution of residuals, or correct specification of random effects structure); in all instances of linear models employed here, at least some of these assumptions were violated. This was not the case with the GAMM, whose assumptions were all met to a satisfactory degree. Additionally, when compared to the models generated by linear modelling approaches, the GAMM explained more of the observed variance, as indicated by a higher R^2^ value compared to that of the ANOVA model, and diagnostic evaluation supported a better model fit.

It should be noted that, while the current study only focused on comparing the GAMM approach with commonly used ANOVA and GLM approaches, these are not the only approaches used for the analysis of VMR-derived data. For instance, Liu et al., 2017 used a generalised linear mixed model (GLMM) approach, in which burst duration (defined as the fraction of frames per second exceeding a movement threshold) were binarized prior to modelling. Specifically, measures were collapsed into zero and non-zero values, coded as movement presence [1] or movement absence [0]. This transformation ultimately means that a single binary indicator could represent, and be treated the same as, seconds with low or high activity. The authors, however, acknowledge that their method cannot capture larval displacement or movement magnitude and recommend pairing it with another statistical method such as Hotelling’s T-squared test (Liu et al., 2015). Another limitation noted by Liu et al. (2017) was that the model’s terms are insufficient to represent nonlinear patterns (across time; temporal aggregation masks neurobehavioural signatures) in activity and recommend smoothing spline ANOVA as a complementary method. The GAMM approach we adopted addresses both of these limitations simultaneously, retaining continuous activity while flexibly modelling nonlinear time dynamics within a mixed-effect framework. Consequently, application of a GAMM provides a richer representation of real locomotor phenotypes and potentially improves the sensitivity with which to detect neurobehavioral effects.

In another study, Gauthier and Vijayan (2018) used a Lorentzian and Ricker-beta function as a flexible time-response curve to describe locomotion after a single light pulse stimulus. This elegant approach was used to describe the activity profile after the light stimulus in an asymmetric way, enabling the authors to capture high-definition information about activity during the excitatory and relaxation periods following the light stimulus. While it would be possible to treat every light/dark transition during the VMR as a separate pulse with distinct excitation and relaxation periods, we argue that, where there is an alternation of sustained light and dark periods, GAMMs offer more flexibility and allow for the statistical analysis of the overall effect of the lighting changes across the duration of the trial, rather than focusing on specific time periods. This is particularly important where chemical exposure could be having a (de)sensitising effect on the animal’s responsiveness to light-dark transitional stimulation.

GAMMs are not the only option available for analysis of non-linear time series data: other options include, among others, generalised estimated equations (GEEs), Bayesian hierarchical GAMs, and state-space models. The current study utilised GAMMs as we believe they present a good balance between flexibility of analysis (i.e., the smoothing parameter is dictated by the data, and not the user, as would be the case for GEEs) and accessibility (compared to approaches which require an advanced understanding of Bayesian inference, or the latent/observed process separation).

The results presented here were obtained after the analysis of data normalised against the behavioural response of the solvent control treatment group. VMR data normalisation is often undertaken to reduce variability induced by systematic factors such as well effects in well plates, and batch-to-batch or time of the day variability (Xie et al., 2019; Hillman et al., 2024b); additionally, it is often used as a pre-processing step when data are analysed using approaches which assume a normal distribution of residuals (such as ANOVA or mixed effects linear models) (Liu et al.,2015; Liu et al., 2017; Xie et al., 2019). Here, data were normalised to allow for direct comparison to such statistical approaches. Given that we subtracted a constant from all groups (i.e., the average of movement across the duration of the trial), absolute variance within each group is largely preserved. It should, however, be noted that normalisation may potentially affect model outcomes, especially if experimental groups differ in their baseline variance and not just their mean (i.e., if they exhibit heteroskedasticity).

One limitation of using the GAMM for analysing locomotion data is that it focuses on distance-based measures and does not consider kinematic endpoints such as speed. Speed is a complementary aspect of motor control and may reveal further chemically-induced neurobehavioral impacts and could in practice be analysed using a GAMM. There are a number of other behavioural endpoints which could also be examined (e.g., those mentioned by Rowson et al., 2025: habituation, startle response and range of activity), and future work should evaluate how non-linear mixed models perform across multidimensional behavioural endpoints, eventually enabling richer phenotypic profiling, and interpretation of the VMR and other neurobehavioral endpoint assays. In fact, with continued assay standardisation and congruent demonstration of cross-laboratory reproducibility, the VMR has the potential to have a more expansive role in elucidating behavioural endpoints for assessing the effects of neuroactive chemicals, including potential applications in environmental risk assessments or in support of phenotypic neuroactive drug screening programmes.

The potential of the VMR assay is further strengthened by ethical and practical advantages: allowing testing to be undertaken at the pre-feeding, pre-protection larval life phase under current United Kingdom and EU animals in science regulations, providing non-invasive testing of sensitive behavioural effects for early hazard identification, thus minimising animal suffering (Thoré et al., 2021), and enabling the reduction (as well as refinement) of animal use in research (Russell and Burch, 1959) i.e., each animal can also potentially be employed across multiple behavioural assays or repeated measures, maximising data acquisition per organism. Taken together, methodological advances that improve analytical robustness and reproducibility will be critical for positioning behavioural assays as credible and scalable tools within comparative and translational toxicological and pharmacological research.

In conclusion, we present a generalised additive mixed modelling (GAMM) approach as a sensitive and rigorous statistical method for resolving the temporal complexity of high-throughput locomotor phenotyping larval zebrafish in the VMR assay, which is being used widely to assess the behavioural effects of neuroactive chemicals. By explicitly modelling non-linear behavioural trajectories within the VMR assay, this approach preserves the structure of time-series data while enabling interpretable treatment contrasts that conventional linear frameworks (e.g., ANOVAs and GLMs) fail to resolve. Application of the model to a VMR dataset examining the effects of the antipsychotic drug clozapine on larval zebrafish (4 dpf) generated a coherent concentration-response profile that revealed a behavioural signature characterised by hypolocomotion differentially expressed depending on lighting status. These findings illustrate how pharmacological effects can emerge as multidimensional shifts in behavioural state, rather than simple suppression (or indeed elevation) of generalised locomotor activity.

Importantly, the GAMM method is not restricted to the specific compound or behavioural endpoint examined here. Although this study focused on distance travelled and the antipsychotic clozapine as an illustrative case, the same modelling strategy can also be extended to other VMR datasets, with different neuroactive compounds expected to produce distinct behavioural signatures reflecting their mechanisms of action. Likewise, broader classes of behavioural time-series (including additional kinematic and spatial features; endpoints such as thigmotaxis, speed, acceleration, bout frequency and structure, freezing, and measures of habituation) capture different aspects of locomotor function and may exhibit different temporal patterns and sources of variability, therefore further refining phenotypic characterisation. Consequently, while the general modelling framework presented here is readily transferable across compounds, endpoints, assays, and laboratories, the precise GAMM specification (including smooth structures, distributions, random effects and correlation structures) should be tailored to the statistical characteristics of the behavioural measure under investigation rather than blanket applied (copy-and-paste approach).

Future studies incorporating multiple behavioural endpoints and chemically diverse compounds will further establish the generality and flexibility of this analytical approach for behavioural phenotyping. As analytical standardisation develops alongside assay harmonisation, automated locomotor phenotyping has the capacity to mature into a sensitive, scalable platform for detecting neurobehavioural disruption. Because many chemicals exert effects on the conserved central nervous system and thus neural function, behavioural readouts offer a uniquely integrative window into potential adverse effects. In this context, statistically robust modelling of behavioural dynamics is essential for translating higher-throughput assays into credible [chemical] screening tools. The approach presented here strengthens the interpretability, reproducibility, and cross-study comparability of VMR data, supporting the further evaluation of larval locomotor analysis for chemical risk assessment. With continued methodological refinement and broader validation, such frameworks could aid the development of standardised test guidelines by providing mechanistically informative, statistically rigorous and quantitatively robust (repeatable) indicators of neuroactive change.

## Data Availability

The clozapine datasets and corresponding code used for all analyses are publicly available on Figshare (10.6084/m9.figshare.31573078). Additionally: Supplementary Material (GAMM Supplementary Results. docx) and Supplementary Table (Supplementary_Table_1. xlsx10.6084/m9.figshare.31573078.
