# Supplementary material for: Refining methodological approaches for analysing higher-throughput locomotor behaviour, using generalized additive mixed models
Source: Front Toxicol. 2026 Aug 21;8:1891859. doi: 10.3389/ftox.2026.1891859 (PMC13541960; doi:10.3389/ftox.2026.1891859)
Supplement: Supplementary file 1 [file Supplementaryfile1.docx]

# **GAMM SUPPLEMENTARY INFORMATION**

## **Supplementary Methods**

### **GAMM specification**

The GAMMs included the total distance travelled (in mm), the lighting regime, the treatment, and a smooth for time for each level of the lighting condition; the nested random effects were individual fish ID to account for any differences between individual larvae, and trial plate ID to account for any variation between different experimental effects. Comparing the variance components estimates for each of the random factors included in the model suggested that both factors explain little residual variance: random intercepts for individual fish were associated with a typical multiplicative deviation of approximately 0.6% from the population mean of total distance moved (standard deviation on the log scale = 0.0056), while random intercepts for trial were associated with a typical deviation of approximately 3.4% (standard deviation on the log scale = 0.034), indicating that trial-to-trial variation was a more substantial source of unexplained variability than fish-to-fish variation (for the exact numbers and calculations, please see the relevant code file). Random effect structure was further verified through comparison of models with nested random effects using AIC, after refitting with Maximum Likelihood (to allow for comparison of models with different random effects structures). Nested model comparison suggested that the inclusion of trial as a random factor significantly increased the fit of the model (ΔAIC = 57 compared to the full model, in favour of the model including trial as a random factor), while the inclusion of individual fish ID had no effect on the model fit (ΔAIC = 0 compared to the full model), suggesting that, while trial should be retained, fish ID is not improving data interpretation. Given, however, that this dataset contains repeated measurements from the same individuals, individual fish ID was retained, as removing it would lead to pseudoreplication, and affect the effective sample size.

The number of knots (k) was validated through k-fold model cross-validation. Briefly, the dataset was divided into 5 equally sized groups (Hijmans 2012) in a way which allows for data from each fish to remain intact; afterwards, a percentage of randomly selected observations were used to run the model and the remaining were used for model validation, iteratively for each fold (5-fold validation). We compared the mean squared error (MSE) for models with k=10, 15, 20, 25, and 30, and found that the average MSE for increasing values plateaued around k=15 (MSE= 1056.678) and k=20 (1056.345), with negligible improvement for values over k=20 (k=30: MSE=1056.265). Basis-dimension model checks (Wood, 2019) confirmed that both k=20 and k=30 were sufficient (k-index around 1, and p > 0.05 in both cases), with effective degrees of freedom below k` at both settings, indicating robustness across a range of k-values ranging from 20-30 (for more information on the 5-fold validation, see the relevant code file). We retained k=30 in the final model presented here as a conservative choice.

### **Outlier removal validation**

To ensure that the removal of outliers prior to data analysis did not affect the findings reported here, we carried out a validation using the whole normalised dataset (including the outliers), and applying the exact GAMM approach already described (i.e. maintaining the same model structure and number of knots). This analysis yielded similar results to the one of the outlier-filtered dataset, with a statistically significant interaction between the lighting conditions and treatment (clozapine concentration) (F=106.66, p<0.001). *Post hoc* contrasts showed similar tendencies between the two analysis, with only some deviations in conditions where contrasts did not reach statistical significance (see Table S4).

**Table S1. Summary of the different types of models used for the analysis of the movement data acquired during the Visual Motor Response (VMR) assay.** In each instance we present the optimal model structure alongside any notes, a summary of the diagnostic plots, the adjusted R^2^ value (as a measure of the total variation explained by the model in each instance), and the key findings of each model. For each model, we also present the core assumptions, and note whether they were violated (in bold) or not. While the quality of fit varied between the different models, they all pointed towards a statistically significant interaction between treatment, lighting conditions, and time.

| **Model** | **Structure** | **Structure notes** | **Diagnostic plots** | **Adjusted R^2^** | **Main findings** | **Model assumptions (and possible violations)** |
| --- | --- | --- | --- | --- | --- | --- |
| **ANOVA/ linear model** | Distance ~ Treatment * Light + Time | Preliminary examination of partial residuals for Time suggests non-linearity.  Technically an ANCOVA if we consider Time a continuous variable and not a factor where each minute is a different level). | Q-Q plot: standardised residuals deviate in the lowest and highest theoretical quartiles | 0.5274 | Significant Treatment * Light * Time interaction (F_(5, 8196)_=9.054, p<0.001). | - **Independence of observations (violated, due to repeated measures)** - **Normality of residuals (violated – deviations in the lowest and higher quartiles)** - Homoscedasticity (borderline) - Interval or ratio-scaled dependent variable (not violated) - **Linearity (violated – non linear relationship between Time and the outcome variable).** - **Homogeneity of regression slopes (if Time is treated as a continuous variable) (violated – significant interaction between Time * Treatment * Light)** |
| **Linear Mixed Effects Model (LMER)** | Distance ~ Treatment * Light * Time + (1\|Fish) | Random effect structure chosen using AIC. Given the fact that this is also a linear model, the non-linearity issue persists. | Q-Q plot: standardised residuals deviate in the lowest and highest theoretical quartiles. Some heteroskedasticity. | 0.539 | Significant Treatment * Light * Time interaction (F_(5, 7920)_=10.855, p<0.001). | - **Linearity (violated – non linear relationship between Time and the outcome variable).** - **Normality of residuals (violated – deviations in the lowest and higher quartiles)** - Normality of random effects (not violated) - Homoscedasticity (borderline) - Independence conditional on random structure (not violated) - No multicollinearity (not violated) - Correctly specified random effect structure (not violated) |
| **Generalised Linear Model (GLM)** | Distance ~ Treatment * Light * Time;  Gaussian family, inverse link function, after adding 0.0000001 to each value to ensure positive values only. | Family and link function chosen based on AIC values and diagnostic plots. | Some deviation of the residuals on the Q-Q plot. Some evidence of heteroskedasticity. | 0.542 | Significant Treatment * Light * Time interaction (F_(5, 8196)_=9.496, p<0.001). | - **Independence of observations (violated, due to repeated measures)** - Correct distributional family (not violated - validated) - Correct link function (not violated – validated) - Linearity on the link scale (not violated) - No multicollinearity (not violated) - Correct mean-variance relationship (no unmodeled overdispersion) (not relevant for Gaussian distributions) |
| **Generalised Linear Mixed Model (GLMER)** | Distance ~ Treatment * Light * Time + (1/Trial/Fish);  Gaussian family, inverse link function, after adding 0.0000001 to each value to ensure positive values only. | Family and link function, as well as random effect structure based on AIC and diagnostic plots. All GLMERs either failed to converge or gave singularity warnings. | Singularity warnings make this type of model unreliable. | Singularity warnings make this type of model unreliable. | Singularity warnings make this type of model unreliable. | - Correct distributional family (not violated - validated) - Correct link function (not violated – validated) - Linearity on the link scale (not violated) - Independence conditional on random effects (not violated) - Normality of random effects (not violated) - No unmodeled overdispersion - **Correctly specified random-effects structure (violated – the inclusion of appropriate random effects structure led to singularity warnings or failure to converge)** - No multicollinearity (not violated) - **Sufficient number of random-effect levels (borderline as indicated by singularity issues)** |
| **Generalised Additive Mixed model (GAMM)** | Distance ~ smooth(Time, by Light) + Light * Treatment + (1\|Fish, 1\|Trial);  Gaussian family with log link function, k=30 | Family and link function, as well as random effect structure based on AIC and diagnostic plots. Number of knots was validated through k-fold validation | Some deviation of the residuals on the Q-Q plot. All other residual plots show no issues with model specification. | 0.597 | Significant smooth for Time, dependent on the lighting condition significant (Dark: F_(12.7, 8185)_=116.4, p< 0.001; Light: F_(10.9, 8185)_=31.7, p<0.001). Significant Treatment * Light interaction (F_(10.9, 8185)_=171.06, p<0.001). | - Correct distributional family (not violated - validated) - Correct link function (not violated – validated) - Adequate basis dimension (k) (not violated – validated) - No concurvity (not violated) - Normality of random effects (not violated) - Independence conditional on random effects and smooths (not violated) - Correctly specified random effects and smooth-term structure (not violated - validated) - No residual temporal autocorrelation (not violated) - Homogeneity of variance (not violated) |

## **Supplementary Results**

**Table S2.** **Estimated effect of lighting conditions and clozapine exposure on the total distance travelled, during a standard locomotion assay compared to the solvent control (SC: DMSO 0.1% v/v), for a specific set of time points reflecting the end of light and dark periods.** Data were analysed by ANOVA. Bold denotes statistically significant differences compared to the SC (p values after Bonferroni adjustment). A DWC was included to assess the effect of solvent (DMSO 0.1% v/v) exposure compared to normal culture water and was found not to have an effect.

| **Lighting condition** | **Time (minute)** | **Treatment (μM)** | **Estimate** | **Std. error** | **t-value** | **p-value** |
| --- | --- | --- | --- | --- | --- | --- |
| Dark | 5 | SC |  |  |  |  |
|  |  | 6.25 | 0.053 | 2.578 | 0.02 | 1 |
|  |  | **1** | **14.579** | **2.6** | **5.607** | **<0.001** |
|  |  | **0.5** | **10.941** | **2.594** | **4.218** | **<0.001** |
|  |  | **0.1** | **9.107** | **2.579** | **3.531** | **0.002** |
|  |  | **DWC** | **7.231** | **2.582** | **2.8** | **0.0256** |
| Light | 10 | SC |  |  |  |  |
|  |  | **6.25** | **-23.041** | **2.579** | **-8.934** | **<0.001** |
|  |  | **1** | **-24.704** | **2.574** | **-9.598** | **<0.001** |
|  |  | **0.5** | **-26.772** | **2.58** | **-10.378** | **<0.001** |
|  |  | **0.1** | **-13.339** | **2.578** | **-5.174** | **<0.001** |
|  |  | DWC | 5.658 | 2.601 | 2.175 | 0.1483 |
| Dark | 15 | SC |  |  |  |  |
|  |  | **6.25** | **-9.727** | **1.917** | **-5.074** | **<0.001** |
|  |  | **1** | **9.981** | **1.918** | **5.203** | **<0.001** |
|  |  | **0.5** | **11.618** | **1.913** | **6.073** | **<0.001** |
|  |  | **0.1** | **9.001** | **1.907** | **4.719** | **<0.001** |
|  |  | DWC | 3.826 | 1.911 | 2.002 | 0.2267 |
| Light | 20 | SC |  |  |  |  |
|  |  | **6.25** | **-14.617** | **2.441** | **-5.988** | **<0.001** |
|  |  | **1** | **7.682** | **2.439** | **3.15** | **0.0082** |
|  |  | **0.5** | **11.956** | **2.429** | **4.923** | **<0.001** |
|  |  | **0.1** | **8.948** | **2.423** | **3.694** | **0.0011** |
|  |  | DWC | 2.124 | 2.432 | 0.873 | 1 |
| Dark | 25 | SC |  |  |  |  |
|  |  | **6.25** | **-19.506** | **3.279** | **-5.948** | **<0.001** |
|  |  | 1 | 5.383 | 3.28 | 1.641 | 0.5037 |
|  |  | **0.5** | **12.294** | **3.264** | **3.766** | **<0.001** |
|  |  | **0.1** | **8.896** | **3.255** | **2.733** | **0.0314** |
|  |  | DWC | 0.422 | 3.269 | 0.129 | 1 |
| Light | 30 | SC |  |  |  |  |
|  |  | **6.25** | **-13.198** | **3.256** | **-4.053** | **<0.001** |
|  |  | **1** | **-24.548** | **3.25** | **-7.553** | **<0.001** |
|  |  | **0.5** | **-32.408** | **3.255** | **-9.956** | **<0.001** |
|  |  | **0.1** | **-17.936** | **3.25** | **-5.519** | **<0.001** |
|  |  | **DWC** | **10.874** | **3.271** | **3.325** | **0.0044** |

**Table S3. Estimated effect of lighting conditions and clozapine exposure on the total distance travelled, during a standard locomotion assay compared to the solvent control (SC: DMSO 0.1% v/v), for a specific set of time points reflecting the middle of light and dark periods.** Data were analysed by ANOVA. Bold denotes statistically significant differences compared to the SC (p values after Bonferroni adjustment). A DWC was included to assess the effect of solvent (DMSO 0.1% v/v) exposure compared to normal culture water and was found not to have an effect.

| **Lighting condition** | **Time (minute)** | **Treatment (μM)** | **Estimate** | **Std. error** | **t-value** | **p-value** |
| --- | --- | --- | --- | --- | --- | --- |
| Dark | 3 | SC |  |  |  |  |
|  |  | 6.25 | 2.008 | 2.906 | 0.691 | 1 |
|  |  | **1** | **15.498** | **2.932** | **5.287** | **<0.001** |
|  |  | **0.5** | **10.806** | **2.924** | **3.695** | **0.0011** |
|  |  | **0.1** | **9.128** | **2.907** | **3.14** | **0.0085** |
|  |  | **DWC** | **7.912** | **2.911** | **2.718** | **0.0329** |
| Light | 8 | SC |  |  |  |  |
|  |  | **6.25** | **-24.025** | **2.908** | **-8.262** | **<0.001** |
|  |  | **1** | **-24.72** | **2.902** | **-8.518** | **<0.001** |
|  |  | **0.5** | **-26.208** | **2.909** | **-9.011** | **<0.001** |
|  |  | **0.1** | **-12.879** | **2.907** | **-4.43** | **<0.001** |
|  |  | DWC | 5.136 | 2.933 | 1.751 | 0.3997 |
| Dark | 13 | SC |  |  |  |  |
|  |  | **6.25** | **-7.771** | **1.862** | **-4.174** | **0.0002** |
|  |  | **1** | **10.901** | **1.866** | **5.84** | **<0.001** |
|  |  | **0.5** | **11.482** | **1.862** | **6.166** | **<0.001** |
|  |  | **0.1** | **9.022** | **1.856** | **4.862** | **<0.001** |
|  |  | DWC | 4.507 | 1.858 | 2.426 | 0.0765 |
| Light | 18 | SC |  |  |  |  |
|  |  | **6.25** | **-19.104** | **1.853** | **-10.311** | **<0.001** |
|  |  | **1** | **-24.642** | **1.849** | **-13.329** | **<0.001** |
|  |  | **0.5** | **-29.026** | **1.853** | **-15.666** | **<0.001** |
|  |  | **0.1** | **-15.177** | **1.849** | **-8.207** | **<0.001** |
|  |  | **DWC** | **7.744** | **1.865** | **4.152** | **0.0002** |
| Dark | 23 | SC |  |  |  |  |
|  |  | **6.25** | **-17.55** | **2.921** | **-6.008** | **<0.001** |
|  |  | 1 | 6.303 | 2.92 | 2.158 | 0.1547 |
|  |  | **0.5** | **12.159** | **2.907** | **4.183** | **<0.001** |
|  |  | **0.1** | **8.917** | **2.899** | **3.076** | **0.0105** |
|  |  | DWC | 1.103 | 2.911 | 0.379 | 1 |
| Light | 28 | SC |  |  |  |  |
|  |  | **6.25** | **-14.183** | **2.9** | **-4.891** | **<0.001** |
|  |  | **1** | **-24.564** | **2.894** | **-8.487** | **<0.001** |
|  |  | **0.5** | **-31.844** | **2.899** | **-10.986** | **<0.001** |
|  |  | **0.1** | **-17.476** | **2.894** | **-6.039** | **<0.001** |
|  |  | **DWC** | **10.352** | **2.912** | **3.555** | **0.0019** |

**Table S4. Estimated effect of lighting conditions and clozapine exposure on the total distance travelled, during a standard locomotion assay compared to the solvent control (SC: DMSO 0.1% v/v), using non-outlier-filtered data.** Data were analysed by a generalised additive mixed model (GAMM). Bold denotes statistically significant differences compared to the SC (p values after Dunnett’s adjustment). A DWC was included to assess the effect of solvent (DMSO 0.1% v/v) exposure compared to normal culture water and was found not to have an effect. The use of a non-outlier filtered version of the dataset gave very similar results to the ones obtained from performing the same analysis on an outlier-filtered dataset (see Table 2).

| **Lighting condition** | **Treatment (µM)** | **Estimate** | **Std. error** | **t-value** | **p-value** |
| --- | --- | --- | --- | --- | --- |
| Light | SC |  |  |  |  |
|  | **6.25** | **-0.2587** | **0.0497** | **-5.205** | **<0.001** |
|  | **1** | **-0.3755** | **0.0511** | **-7.345** | **<0.001** |
|  | **0.5** | **-0.4806** | **0.0522** | **-9.206** | **<0.001** |
|  | **0.1** | **-0.2044** | **0.0495** | **-4.132** | **0.002** |
|  | DWC | 0.0306 | 0.0480 | 0.637 | 0.9108 |
| Dark | SC |  |  |  |  |
|  | 6.25 | -0.1081 | 0.0447 | -2.416 | 0.0663 |
|  | 1 | 0.0650 | 0.0447 | 1.467 | 0.4392 |
|  | 0.5 | 0.1011 | 0.0447 | 2.286 | 0.0914 |
|  | 0.1 | 0.0794 | 0.0447 | 1.794 | 0.2588 |
|  | DWC | 0.0369 | 0.0447 | 0.832 | 0.8273 |

## **References**

Hijmans, R. J. (2012). Cross‐validation of species distribution models: removing spatial sorting bias and calibration with a null model. *Ecology*, *93*(3), 679–688. <https://doi.org/10.1890/11-0826.1>

Wood, S. N. (2019). mgcv: mixed GAM computation vehicle with automatic smoothness estimation (version 1.8-31).
